# Clinical effects of IPCH after the surgery for ovarian cancer at the middle or advanced stage and its impacts on tumor markers and immune functions

**DOI:** 10.12669/pjms.40.9.8720

**Published:** 2024-10

**Authors:** Heyue Li, Yan Zhang, Hong Tang, Linxia Li

**Affiliations:** 1 Heyue Li, Division of Urological and Reproductive Medicine, Shanghai 7^th^ People’s Hospital, Affiliated to Shanghai University of Traditional Chinese Medicine, Shanghai, 200137, China; 2 Yan Zhang, Division of Urological and Reproductive Medicine, Shanghai 7^th^ People’s Hospital, Affiliated to Shanghai University of Traditional Chinese Medicine, Shanghai, 200137, China; 3 Hong Tang, Division of Urological and Reproductive Medicine, Shanghai 7^th^ People’s Hospital, Affiliated to Shanghai University of Traditional Chinese Medicine, Shanghai, 200137, China; 4 Linxia Li, Division of Urological and Reproductive Medicine, Shanghai 7^th^ People’s Hospital, Affiliated to Shanghai University of Traditional Chinese Medicine, Shanghai, 200137, China

**Keywords:** Intraperitoneal chemohyperthermia, Advanced stage ovarian cancer, Clinical effect, Tumor marker, Immune function

## Abstract

**Objective:**

To evaluate the clinical effects of intraperitoneal chemohyperthermia (IPCH) in the treatment of postoperative patients with advanced stage ovarian cancer, and its impacts on tumor markers and immune functions.

**Methods:**

This is a retrospective study. One hundred and twenty patients with advanced stage ovarian cancer received in Shanghai 7th People’s Hospital Affiliated to Shanghai University of Traditional Chinese Medicine from May 10, 2022 to June 10, 2023 were selected and randomly divided into control and study groups (n=60 each group). Patients in the control group were administered routine intravenous chemotherapy, the study group underwent IPCH besides routine intravenous chemotherapy based on the control group. Comparatively analyzed the clinical effects, adverse reaction occurrence rates, and variations in relevant observation targets, the tumor marker after and before the treatment.

**Results:**

Total efficacy in the study and control groups are proved to be with statistically significant differences. The occurrence of adverse reactions proved that no significant differences is seen between both groups. After the treatment, CEA, CA19-9, and CA125 levels in patients of the study group are apparently lower than those in the control group, showing statistically significant differences; expression levels of CD3^+^, CD4^+^ and CD4^+^/CD8^+^, in the study group were enormously above those in the control group after the treatment, with statistically significant differences.

**Conclusions:**

IPCH has the potential to effectively enhance comprehensively clinical therapeutic effects among postoperative patients with ovarian cancer, significantly improve patients’ immune states, and evidently reduce expression levels of various tumor markers.

## INTRODUCTION

Ovarian cancer is a common type of malignant tumor in the gynecological reproductive system.^1^ Early symptoms in most patients cannot be easily detected in clinics. Generally, it is manifested as malignant ascites at the time of onset. Considering that ovarian cancer has a low early diagnosis rate, patients’ condition can be dramatically severe and those developing advanced ovarian cancer also occupy a large proportion of the total population. Some research^2^ reported that 70% of patients with ovarian cancer are at a late stage when confirmed, which produces high mortality. Besides, mortality of ovarian cancer takes second place among gynecological cancers across the world.^3^ Nowadays, radical cytoreductive surgery and postoperative adjuvant chemotherapy have become standard therapeutic schedules for ovarian cancer.^4^ Commonly used chemotherapeutic drugs in clinics for postoperative patients are platinum-based or taxol.^5^ In the context of adopting routine intravenous chemotherapy, drugs are present in the blood, so it is hard to deliver these drugs to abdominal and pelvic cavities. However, the postoperative recurrence is relatively high due to remaining free tumor cells existing after the surgery performed for advanced ovarian cancer patients.^6^ In this case, the patients may eventually die of tumor progression. Besides, fine granular neoplastic foci cannot be easily excised through operation. Therefore, providing patients with targeted chemotherapy after the surgery is believed to have great clinical significance. Moreover, ovarian cancer is sensitive to chemotherapy, and its disseminated and metastatic foci are merely confined to the abdominopelvic cavity. With continuous theoretical and practical advances in tumor diagnosis and treatment, intraperitoneal chemohyperthermia (IPCH) is gradually applied in ovarian cancer treatment and shows certain superiorities.^7^ In this study, IPCH combining routine intravenous chemotherapy in the clinical treatment of patients with advanced stage ovarian cancer, to explore the clinical effects of intraperitoneal chemohyperthermia (IPCH) in the treatment of postoperative patients with advanced stage ovarian cancer, and its impacts on tumor markers and immune functions.

## METHODS

One hundred and twenty patients with advanced stage ovarian cancer received in Shanghai 7th People’s Hospital Affiliated to Shanghai University of Traditional Chinese Medicine from May 10, 2022 to June 10, 2023 were selected and randomly divided into control and study groups, with each group of 60 cases. Through comparison, general data of patients in both groups show no significant differences, and inter-group differences are comparable (Table-I).

**Table-I:** Comparison of general data in the study and control groups (*χ̅*±*S*) n=60.

Targets	Study group	Control group	t/χ^2^	P
Age	58.35±7.68	57.62±6.58	0.56	0.58
Pathological patterns				
Serous carcinoma	32(%)	34(%)	0.13	0.71
Mucous carcinoma	18(%)	17(%)	0.04	0.84
Adenocarcinoma	7(%)	5(%)	0.37	0.54
Others	3(%)	4(%)	0.15	0.70
Tumor sites			0.31	0.58
Left	36(%)	33(%)		
Right	24(%)	27(%)		
Clinical stages			0.43	0.51
III	48(%)	45(%)		
IV	12(%)	15(%)		
KPS	74.86±7.41	75.03±7.50	0.12	0.90
BMI	23.46±3.18	22.89±3.82	0.89	0.37

*P>0.05.*

### Ethical Approval:

The study was approved by the Institutional Ethics Committee of Shanghai 7th People’s Hospital, Affiliated to Shanghai University of Traditional Chinese Medicine (No.: 2023-7th-HIRB-048; date: July 05, 2023), and written informed consent was obtained from all participants.

### Inclusion criteria:

- Patients with advanced stage tumors (III-IV stage);
- Patients undergoing cytoreductive surgery and pathologically diagnosed with ovarian cancer;
- Patients aged below 75, and the expected survival time ≥ 6 months;
- Patients with KPS function rating >60 points;
- Patients with no myelosuppression or hematopoietic dysfunction;
- Patients whose family members are willing and able to cooperate in the whole course of this study, and showing preferable treatment compliance;
- Patients having no contraindications for the drugs used in this study.

### Exclusion criteria:

- Patients attacked by recurrent ovarian cancer;
- Patients with ovarian cancer accompanied by severe infectious diseases, immune diseases, and severe dysfunction of vital organs;
- Patients with ovarian cancer combining malignant tumors in other systems;
- Patients having difficulty in continuing the treatment because of serious toxic and side effects;
- Patients with mental diseases or cognitive abnormalities, and failing in cooperating with and fulling the study;
- Patients taking medicine recently that may affect this study, such as immunosuppressors and hormones.

Before treatment, routine examinations were conducted for patients in both groups, such as blood cell analysis, liver function examination, and renal function examination. For exceptional targets detected, improvements were made. Hydration was performed one day before treatment, and patients in the control group were administered routine intravenous chemotherapy. Specifically, one day after the surgery, an intravenous drip of taxol was given at a dose of 175 mg/m^2^, before which, 5 mg of tropisetron was intravenously injected to relieve gastrointestinal reactions. Two days after the surgery, intravenous drip of carboplatin was performed at a dose of 100 mg/m^2^. A single course of treatment lasts for 21 days, and the treatment continued for six courses.

After the operation, patients in the study group received IPCH combining the routine intravenous chemotherapy the same as that selected for the control group, which is elaborated below. A silicon tube indwells during the surgery. On the first day after the surgery, IPCH was performed. In this process, the temperature was set at 43°C. Firstly, the patients were administered 0.9% normal saline at 43°C to conduct peritoneal irrigation, which was followed by IPCH using cis-platinum (dosage: 30 mg/m^2^) and 3,000-4,000 ml of 0.9% normal saline. This procedure was repeated for three days. In the whole course of IPCH, close attention should be paid to changes in the total volume of abdominal perfusion fluids. Likewise, a single course of treatment lasted for 21 days, and the treatment covered six courses. Both group were followed-up time for six months, and case data collection ceased in June 2023.

### Observation targets:

Evaluation of therapeutic effects.^8^ Complete remission (CR) signifies that the lesion disappears, and expression levels of tumor markers restore to their normal values, which lasts for at least four weeks. Partial remission (PR) means that the total length of a lesion is lowered by 30% and above, and the concentration of tumor markers drops as well, which lasts for at least four weeks. Stable disease (SD) signifies that the sum of tumor lengths increases by no more than 20%, and no prominent changes are found in expression levels of tumor markers although the target lesion does not shrink. Progression of disease reflects that the sum of baseline focal lesion lengths is raised by over 20%, and expression levels of tumor markers are up-regulated. Here, the overall response rate (RR)=CR+PR%.

Evaluation of adverse reactions to drugs. Adverse drug reactions of patients in both groups were recorded, including their gastrointestinal reactions, myelosuppression, liver and kidney function impairment, drug allergy, and joint pain.

Comparative analysis of tumor markers. Before and after the treatment, morning fasting blood was drawn respectively to test concentrations of tumor markers such as CA199, CEA, and CA125, and comparatively analyze their differences among patients in both groups.

Comparative analysis of immune functions of participants. Before and after the treatment, venous blood was drawn from patients to test immune molecules CD3^+^, CD4^+^, CD8^+^, and CD4^+^/CD8^+^to comparatively analyze the variations in those targets before/after the treatment can be.

### Statistical analysis:

Data statistics are fulfilled using SPSS 20.0, and relevant measurement data are expressed in(*χ̅*±*S*). While independent samples T-test is adopted for inter-group data analysis, a pairwise T-test is carried out for intra-group data analysis. χ^2^ test is selected for the comparison of respective rates. In the event of P<0.05, it is deemed that the differences are statistically significant.

## RESULTS

Comparative analysis results of treatment effects are shown in Table-II Overall response rates of the study and control groups reach 90% and 72%, respectively. As can be seen, the study group outperforms the control group, with statistically significant differences (p=0.01). Adverse reaction occurrence in the study and control groups register at 27% and 15%, respectively, showing no significant differences (p=0.12). Table-III.

**Table-II:** Treatment effect comparison results between the study and control groups (*χ̅*±*S*) n=60.

Groups	CR	PR	NC	PD	RR
Study	34	20	4	2	54(90%)
Control	26	17	9	8	43(72%)
χ^2^					6.51
P					0.01

*P<0.05.*

**Table-III:** Adverse drug reaction comparison results between the study and control groups (*χ̅*±*S*) n=60.

Groups	Gastrointestinal reactions	Myelosuppression	Renal dysfunction	Allergy	Hepatic dysfunction	Joint pain	Occurrence rates
Study	4	3	3	1	3	2	16 (27%)
Control	0	2	1	2	2	1	9 (15%)
χ^2^							2.48
P							0.12

*p*>0.05.

Expression levels of CEA, CA19-9, and CA125 show no significant differences before the treatment for patients in both groups (p>0.05). After treatment, however, expressions of the above targets are prominently lowered in the study group compared with the control group; and their differences are statistically significant, p=0.00 (Table-IV).

**Table-IV:** Comparison results of tumor marker concentrations between the study and control groups (*χ̅*±*S*) n=60.

Study targets	Observation timepoints	Study group	Control group	T	p
CEA (mg/ml)	Before treatment	8.24±3.17	8.43±2.94	0.34	0.73
	After treatment *	4.26±0.23	4.72±1.12	3.12	0.00
CA19-9 (U/ml)	Before treatment	12.03±3.14	12.31±3.61	0.43	0.65
	After treatment *	3.82±1.03	5.18±1.27	6.44	0.00
CA125 (U/ml)	Before treatment	5.17±0.24	5.04±0.72	1.33	0.19
	After treatment *	3.10±0.13	3.25±0.22	4.87	0.00

* *p* <0.05.

No significant differences are found in the expression levels of CD3^+^, CD4^+^, CD8^+^_,_ and CD4^+^/CD8^+^ before patients in both groups receive any treatment (p>0.05). After treatment, their expression levels in the study group are apparently above those in the control group, with statistically significant differences (p=0.00). With respect to CD8^+^, its variations before and after the treatment are proved to be insignificant as far as patients in both groups are concerned (p>0.05) as shown in Table-V.

**Table-V:** Comparison results of T-lymphocyte subpopulation concentrations between the study and control groups (*χ̅*±*S*) n=60.

Targets	Observation timepoints	Study group	Control group	t	p
CD3+ (%)	Before treatment	44.67±8.22	44.53±8.74	0.09	0.93
	After treatment *	50.53±8.64	45.86±8.53	2.98	0.00
CD4+ (%)	Before treatment	24.52±6.62	25.01±6.37	0.41	0.68
	After treatment *	29.96±6.48	26.77±6.81	2.63	0.00
CD8+ (%)	Before treatment	23.35±5.61	23.18±5.47	0.17	0.86
	After treatment	24.53±5.73	24.38±5.64	0.13	0.79
CD4+/CD8+	Before treatment	1.25±0.27	1.31±0.31	1.13	0.26
	After treatment*	1.84±0.25	1.33±0.31	9.92	0.00

* *p* <0.05.

## DISCUSSIONS

This study empirically proves that IPCH has an overall response rate of 90% in the study group; comparatively, the overall response rate in the control group is 72%. Their differences are of statistical significance (p=0.01). It is reported in a study in the Netherlands (OVIPCH)^9^ that applying IPCH in combination with interval cytoreductive surgery (ICS) for the treatment of Stage-III ovarian cancer is beneficial for progression free survival (PFS) and overall survival (OS) extension. Clearly, IPCH can be used as a new consolidation therapy for ovarian cancer.

As theories and skills of ovarian cancer treatment become increasingly mature, the above problems have been considerably investigated in clinics, and positive progress has been made.^10^ Intraperitoneal chemohyperthermia (IPCH) is a novel method for the treatment of advanced stage ovarian cancer. Gradually, it begins to present certain advantages in the clinical treatment of ovarian cancer.^11^ As for the theoretical basis for IPCH, compared with normal cells, tumor cells are more susceptible to high temperatures.^12^ In other words, tumor cells can be irreversibly damaged when the temperature rises to 43°C, so the activity of such cells can be substantially reduced and the corresponding replication process be extended or even destructed. At high temperature, chemotherapeutics becomes more active and their permeability between tissues and cells is also elevated.^13^ Through peritoneal perfusion, the concentration of chemotherapeutics in local tissues can be significantly boosted for patients, which substantially improves the comprehensive curative effects of chemotherapy.^14^ IPCH combines local chemotherapy with thermal therapy to produce a synergistic effect. Not only is the sensitivity of chemotherapeutics enhanced, but occurrence rates of implantation metastasis and local recurrence are reduced. Without a doubt, IPCH compensates for the defect of intravenous chemotherapy, that is the low concentration of drugs in the abdominal cavity. Moreover, chemotherapeutics in the abdominal cavity is absorbed by the peritoneum and thus delivered to the inferior vena cava and hepatic portal veins, enabling the drug concentration in the liver to be higher than that generated by intravenous chemotherapy. Therefore, IPCH can better prevent hepatic metastases of tumors.

Clinical studies demonstrate that gastrointestinal reaction is an early complication and major adverse response during and after IPCH, which is followed by myelosuppression.^15^ In this study, the adverse reaction of the study group was 27%, while that in the control group is 15%. And, no significant difference is found in the adverse reaction rates of patients in both groups (p=0.12). This is possibly attributed to the fact that chemotherapeutics in local sites of the abdominal cavity is in direct contact with the gastrointestinal tract. Additionally, other adverse reactions such as myelosuppression and renal function impairment remain less severe than those in the control group. This reveals that patients of the study group are preferably IPCH tolerant. As proved in another investigation by Van et al.^16^ it is safe to treat abdominal malignancies using IPCH which keeps the temperature in the abdominal cavity at 43°C, and corresponding complications are tolerable. Both survival rates and quality of patients can be significantly improved, proving that implementing IPCH combined with intravenous chemotherapy for postoperative patients with ovarian cancer is feasible and safe.

Expression levels of tumor markers are important indexes used to evaluate the therapeutic effects of tumor patients.^17^ In terms of various tumor marker targets, they are improved in both groups if compared with those before treatment. Moreover, the targets of tumor markers in the study group are all superior to those in the control group, with statistically significant differences (p=0.00). CA199, CEA, CA153 and CA125 are all effective tumor markers commonly seen in clinics. Variations in these targets can sufficiently reflect specific effects of clinical tumor treatment means, and analyzing these variations is of great clinical significance in identifying postoperative recurrence and metastasis tendencies among patients. Through IPCH, high-temperature chemotherapeutics disrupt the stable state of cancer cells and break down synthetic processes of DNA and proteins in cancer cells, thus ensuring expression levels of associated tumor markers remain at a low level.^18^

Immune function status is also a critical factor in tumor prognosis evaluation.^19^ Hornburg et al.^20^ believed that it is the most sensitive and most effective target in tumor associated disease outcomes. According to this study, concentrations of CD3+, CD4+, and CD4+/CD8+ are all considerably elevated after the treatment. Furthermore, blood in normal tissues and systems of the body, especially the immune system, circulates rather well under actions of hot stress that results in vasodilatation and blood flow acceleration, which further improves immune functions of the body to resist impairment factors.^21^ This may be one of the reasons why the immune functions of the study group are stronger than those in the control group. As the study shows, combining IPCH with intravenous chemotherapy is effective in treating advanced ovarian cancer, and can be continuously promoted and applied in clinics.

### Limitations:

It includes a small sample size, a short follow-up period, and a failure in incorporating the prognosis and survivals of patients. In the future, we will increase the same size, extend the follow-up time, and compare differences in the survivals of patients undergoing diverse therapeutic schedules. Thus, it is expected to more objectively evaluate the clinical effects of the proposed regimen and benefit more patients.

## CONCLUSIONS

IPCH has the potential to effectively and comprehensively enhance the clinical effects of postoperative patients with ovarian cancer, significantly improve their immune state, and tremendously reduce expression levels of various tumor markers without aggravating relevant adverse reactions. Without a doubt, it may be one of the reliable methods for clinical treatment of ovarian cancer.

### Authors’ Contributions:

**HL and YZ** carried out the studies, data collection, drafted the manuscript, are responsible and accountable for the accuracy or integrity of the work.

**HT** performed the statistical analysis and participated in its design.

**LL** participated in acquisition, analysis, or interpretation of data and draft the manuscript.

All authors read and approved the final manuscript.
